# Clinical and laboratory evaluation of patients with SARS-CoV-2 pneumonia treated with high-titer convalescent plasma

**DOI:** 10.1172/jci.insight.143196

**Published:** 2021-03-22

**Authors:** Michele L. Donato, Steven Park, Melissa Baker, Robert Korngold, Alison Morawski, Xue Geng, Ming Tan, Andrew Ip, Stuart Goldberg, Scott Rowley, Kar Chow, Emily Brown, Joshua Zenreich, Phyllis McKiernan, Kathryn Buttner, Anna Ullrich, Laura Long, Rena Feinman, Andrea Ricourt, Marlo Kemp, Mariefel Vendivil, Hyung Suh, Bindu Balani, Cristina Cicogna, Rani Sebti, Abdulla Al-Khan, Steven Sperber, Samit Desai, Stacey Fanning, Danit Arad, Ronaldo Go, Elizabeth Tam, Keith Rose, Sean Sadikot, David Siegel, Martin Gutierrez, Tatyana Feldman, Andre Goy, Andrew Pecora, Noa Biran, Lori Leslie, Alfred Gillio, Sarah Timmapuri, Michele Boonstra, Sam Singer, Sukhdeep Kaur, Ernest Richards, David S. Perlin

**Affiliations:** 1 John Theurer Cancer Center, Hackensack University Medical Center, Hackensack, New Jersey, USA.; 2 Hackensack Meridian Health Center for Discovery and Innovation, Nutley, New Jersey, USA.; 3 Department of Biostatistics, Bioinformatics and Biomathematics, Georgetown University, Washington, DC, USA.; 4 Hackensack University Medical Center, Hackensack, New Jersey, USA.

**Keywords:** COVID-19, Immunotherapy

## Abstract

Here, we report on a phase IIa study to determine the intubation rate, survival, viral clearance, and development of endogenous Abs in patients with COVID-19 pneumonia treated with convalescent plasma (CCP) containing high levels of neutralizing anti–SARS-CoV-2 Abs. Radiographic and laboratory evaluation confirmed all 51 treated patients had COVID-19 pneumonia. Fresh or frozen CCP from donors with high titers of neutralizing Abs was administered. The nonmechanically ventilated patients (*n* = 36) had an intubation rate of 13.9% and a 30-day survival rate of 88.9%, and the overall survival rate for a comparative group based on network data was 72.5% (1625/2241). Patients had negative nasopharyngeal swab rates of 43.8% and 73.0% on days 10 and 30, respectively. Patients mechanically ventilated had a day-30 mortality rate of 46.7%; the mortality rate for a comparative group based on network data was 71.0% (369/520). All evaluable patients were found to have neutralizing Abs on day 3 (*n* = 47), and all but 1 patient had Abs on days 30 and 60. The only adverse event was a mild rash. In this study on patients with COVID-19 disease, we show therapeutic use of CCP was safe and conferred transfer of Abs, while preserving endogenous immune response.

## Abstract 2

Patients with COVID-19 pneumonia receiving high-titer convalescent plasma showed antibody transfer with preservation of endogenous production, and database survival comparison is promising for the early group.

## Introduction

As of December 28, 2020, more than 79 million people around the world have been infected with severe acute respiratory syndrome coronavirus 2 (SARS-CoV-2), and more than 1.7 million have died (1). The human and economic impact, unprecedented in our generation, has mobilized the medical community in search of effective treatment strategies. The angiotensin-converting enzyme 2 (ACE2) is necessary for SARS-CoV-2 to enter human cells (2). The initial phase of the disease occurs when SARS-CoV-2 infects the respiratory epithelial cells. However, in addition to lung tissue, ACE2 expression is found broadly in renal, intestinal, and adipose cells, leading to a wide viral impact on the host (3). Moreover, ACE2 upregulation has been linked to SARS-CoV-2 infections (4). The innate immune response to the viral infection leads to the release of cytokines, and the ensuing cytokine storm results in acute respiratory distress syndrome and multiorgan failure (5). The natural response to viral infections, including coronaviruses, is the production of high-affinity IgG during the adaptive immune response (6). SARS-CoV-2 has been associated with the suppression of this T cell–mediated immune response, bringing into question the quality of the adaptive immunity in severely ill patients (7). Therefore, a therapeutic intervention focused on viral neutralization is a priority.

Convalescent plasma (CCP) as a method of passive immunity transfer has a long history dating to the Spanish flu pandemic of 1918 (8). More recently, CCP was deployed in the management of SARS (9) and Middle East respiratory syndrome (10), with evidence of viral neutralization. CCP therapy in the setting of SARS-CoV-2 infection is currently an active field of investigation (11–23), but information on immune transfer, subsequent endogenous response, and clinical course of patients at different stages of the disease remains incomplete. Furthermore, since the development of neutralizing Ab titers varies among patients who have recovered from coronavirus disease 2019 (COVID-19), CCP is a heterogeneous product of varying potency. In this study, we investigated both the clinical and laboratory parameters characterizing patients treated with high-titer anti–SARS-CoV-2 neutralizing CCP.

## Results

Between April 15, 2020, and June 16, 2020, 52 patients were enrolled. However, 1 patient who had a negative SARS-CoV-2 nasopharyngeal swab reverse transcription PCR (RT-PCR) was considered ineligible. For research purposes across studies, patients with COVID-19 at Hackensack University Medical Center were divided into 3 tracks based on acuity, with track 1 for outpatients, track 2 for patients hospitalized but not requiring positive pressure mechanical ventilation, and track 3 for patients receiving positive pressure mechanical ventilation. Fifty-one patients were treated; 36 patients met criteria for track 2, and 15 patients met criteria for track 3. All 51 patients had radiographic evidence of pneumonia. A substantial proportion of patients in track 2 either were immunocompromised (22%) or had active cancer (19%), as our hospital harbors a cancer center and stem cell transplant program. Demographic and baseline characteristics of patients in tracks 2 and 3, along with patients in our network COVID-19 database, are summarized in Table 1.

Among the 36 patients in track 2, 24 (66.7%) patients were infused with 500 mL liquid fresh irradiated plasma, and 12 (33.3%) patients received 400 mL fresh frozen plasma. Distribution of fresh or frozen plasma was based strictly on availability. The median dose of plasma IgG1–4 infused was 27,537 μg/kg (IQR 21,550–61,408; *n* = 23); 12/36 (33.3%) patients received plasma with viral neutralizing anti–spike protein titers greater than 1:10,000, 22/36 (61.1%) patients received plasma with titers 1:1000–10,000, and 2/36 (5.6%) patients received plasma with neutralizing titers 1:500–1000. The primary endpoint analysis for track 2 showed that patients had an intubation rate of 13.9% (95% CI: 4.7%–29.5%), enough evidence to reject our null hypothesis. Univariate analysis of numerous parameters was performed and is described in Table 2 and Table 3. Older age was associated with an increased risk of intubation. The univariate analysis significance of tocilizumab cannot be ascertained as it was administered to patients with more severe disease. The rates of negative nasopharyngeal swab by RT-PCR on days 10 and 30 after treatment were 43.8% (95% CI: 26.4%–62.3%) and 73% (95% CI: 52.2%–88.4%), respectively. There was only 1 COVID-19–related readmission, and the patient was subsequently discharged.

**Figure 1:**
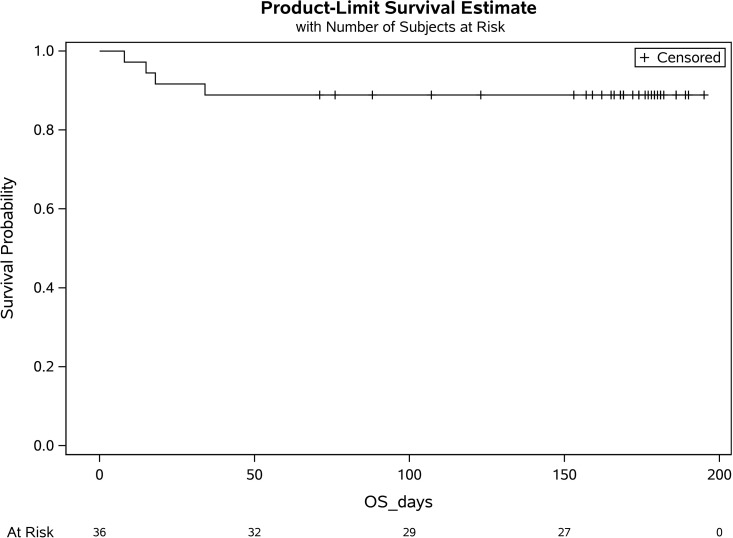
Overall survival for patients nonmechanically ventilated (track 2). BLQ, below limit of quantification; OS, overall survival.

Secondary endpoint analyses for track 2 demonstrated a day-30 survival rate of 88.9% (32/36; 95% CI:73.9%–96.9%; Figure 1). Survival was compared with our network database for hospitalized patients with COVID-19 pneumonia from March 2020 to May 2020. Data fields were selected for age 18 or older, a positive SARS-CoV-2 RT-PCR, and an abnormal chest x-ray or CT scan and excluded for positive pressure mechanical ventilation. A total of 2241 patients met these criteria, with a survival rate of 72.5% (1625/2241; *P* = 0.036). Compared with the database, the track 2 study group was younger, had more patients with active cancer or pregnancy, and had greater supplemental oxygen requirements. There were also more women and more patients receiving remdesivir (Table 1).

Among the 15 patients in track 3, 12 patients (80%) were infused with 500 mL liquid fresh irradiated plasma, and 3 patients received fresh frozen plasma consisting of either 200 mL (1 patient) or 400 mL (2 patients); volume and frozen status were strictly based on availability. The median dose of infused plasma IgG1–4 μg/kg was 38,260 (IQR 33,3076–50,426; *n* = 12); 5/15 patients (33.3%) received plasma with neutralizing anti–spike protein titers greater than 1:10,000, and 9/15 patients (60%) received plasma with titers 1:1000–10,000. The primary endpoint analysis for track 3 showed that patients had a day-30 mortality of 46.7% (7/15; 95% CI:21.3%–73.4%). Based on our study statistical plan, track 3 was closed after 15 patients as the null hypothesis could not be rejected. In a post hoc analysis, the track 3 mortality rate was compared with our network database for hospitalized patients with COVID-19 pneumonia from March 2020 through May 2020. Data fields were selected for a positive SARS-CoV-2 RT-PCR and an abnormal chest x-ray or CT scan, and positive pressure mechanical ventilation. A total of 520 patients met these criteria with a mortality rate of 71% (369/520; *P* = 0.08). Compared with track 3 study patients, database patients were older, and more were receiving invasive mechanical ventilation (Table 1). Secondary endpoint analysis for track 3 study patients showed negative nasopharyngeal swab or endotracheal secretion analysis rates by RT-PCR of 85.7% (95% CI: 42%–100%; *n* = 7) and 100% (95% CI: 63%–100%; *n* = 8) at days 10 and 30, respectively, with a median time from symptom onset to treatment of 15 days (IQR 9–19). There were no readmissions.

**Figure 2:**
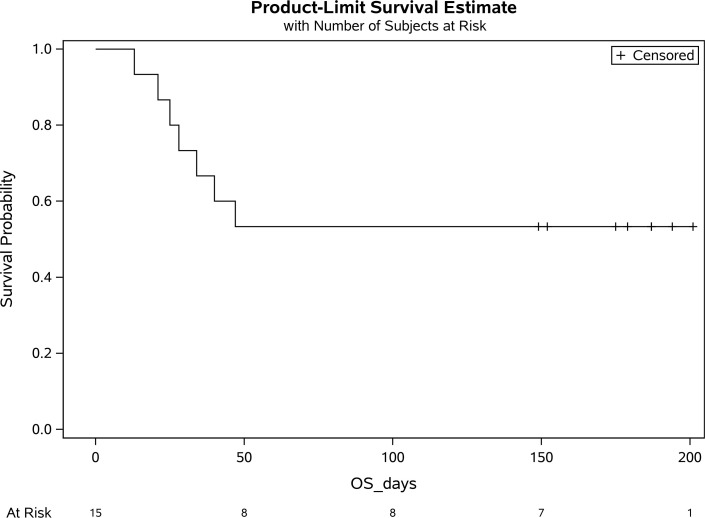
Survival of patients on positive pressure mechanical ventilation (track 3).

A single adverse event occurred for all 51 patients, in which 1 patient developed a grade 2 rash (CTCAE v4.0) for which hydrocortisone 100 mg i.v. was administered once with resolution. Univariate analysis of numerous parameters was performed and is described in Table 2, Table 3 and Table 4. For either track, there was no statistically significant difference in survival, duration of hospitalization, postinfusion antiviral titers, and postinfusion inflammatory markers (C-reactive protein, ferritin, IL-6, and D-dimers) between fresh and frozen plasma, infused plasma IgG subtype (IgA, IgM, IgG1–4) content, or concomitant medications (listed in Table 1). There was also no difference in these endpoints within the ranges of donor IgG antiviral titers used, which were all greater than 1:500 (2 donors) and predominantly greater than 1:1000. The overall survival (OS) plots for each track are shown in Figure 1 and Figure 2. Track 2 and 3 survival comparison with network data is summarized in Table 5.

**Figure 3:**
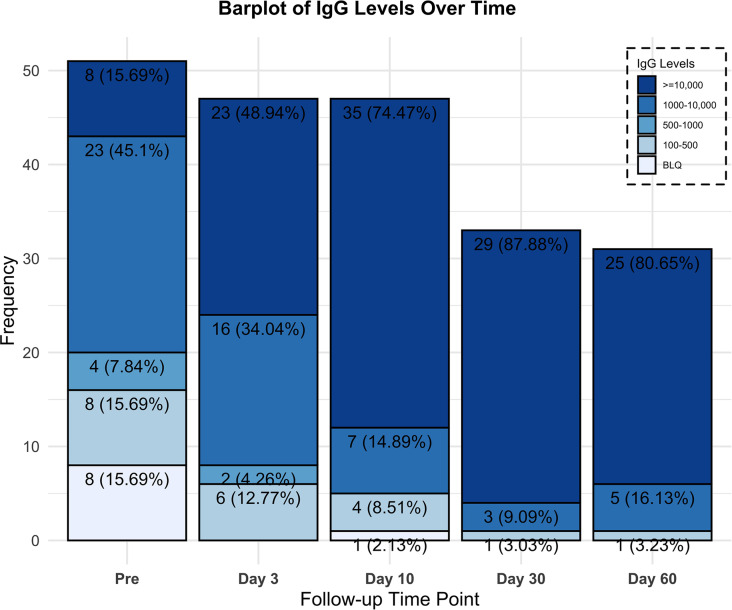
Recipients’ neutralizing Ab titers percentage and frequency over time for all patients.

**Figure 4:**
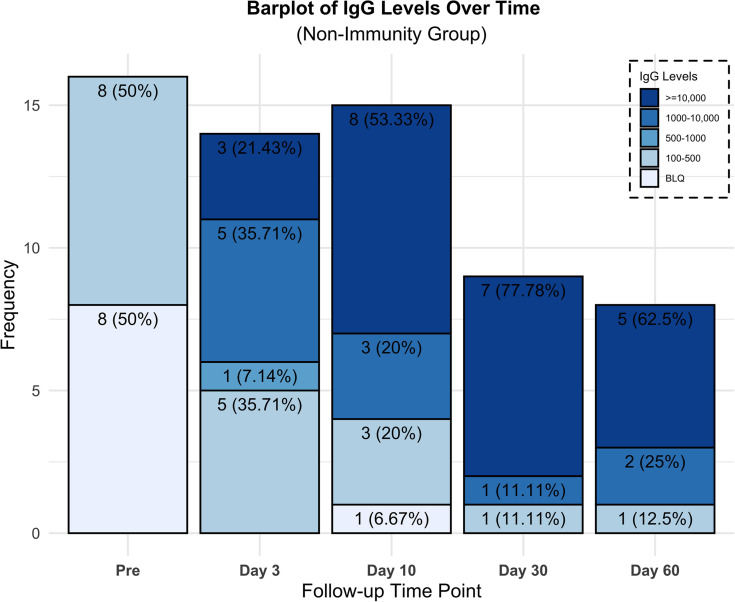
Neutralizing Ab titers percentage and frequency over time for nonimmune or minimally immune patients (≤1:100).

Transfer of immune titers as illustrated in Figure 3 and Figure 4 was evaluated by measuring the recipients’ anti–SARS-CoV-2 neutralizing anti–spike protein recombinant spike receptor binding domain (RBD) titer levels immediately preinfusion and again on day 3. Eight patients (22.2%), all in track 2, had no preinfusion titers, and subsequently all 8 were found to have anti–SARS-CoV-2 neutralizing titers on day 3. One transplant patient on immunosuppression, however, was found to have undetectable titers on day 10. All 15 patients in track 3 had anti–SARS-CoV-2 titers preinfusion, 4/15 patients (27%) greater than 1:10,000, 10/15 patients (67%) 1:1000–10,000, and 1/15 patients (7%) 1:500–1000. However, we observed an increase on day 3 with 12/15 patients (80%) greater than 1:10,000 and 3/15 patients (20%) 1:1000–10,000. All but 1 evaluated patient in the study were found to have neutralizing titers on day 30 (*n* = 33) and all patients on day 60 (*n* = 31; Figure 3).

## Discussion

In this prospective study investigating the therapeutic use of CCP in patients with COVID-19 disease, we showed that the administration of high-titer donor plasma is safe and effectively transfers antiviral titers, while preserving the endogenous development of immunity. The study was conducted at the height of the epidemic in New Jersey, when most patients were hospitalized only if requiring oxygen supplementation. In congruence with this fact, all patients treated had pneumonia. Only 17% of patients concomitantly received remdesivir, allowing for the evaluation of CCP as the sole antiviral agent administered for most patients. Our results showed an intubation rate of 13.9% and for the ventilated patients a day-30 mortality of 46.7%. In a post hoc analysis, the OS of the treated nonmechanically ventilated patients compares favorably with our network database, within the limitations of nonmatched controls. Within the ranges of plasma antiviral titers above 1:1000, we were not able to see a difference in outcome based on titer levels. Frozen plasma was not inferior to fresh plasma. Plasma was infused without adverse events, except for 1 mild rash, to a wide spectrum of recipients, including those who were ventilated, elderly, pregnant, or immunocompromised.

In the search for antiviral therapy, our findings clearly demonstrate the safety of CCP and the passive transfer of antiviral titers. Because the original data from China used fresh liquid plasma (11) and most centers in the United States make use of fresh frozen plasma, the lack of a significant difference between these products is important information. Frozen plasma allows the flexibility of use, as it can be accumulated and rapidly deployed during a viral surge. Since most of the plasma was from donors with titers above 1:1000, we cannot determine a lowest acceptable level. However, we can ascertain within the statistical limits of this study that we need not limit our donor pool to those with the highest titers greater than 1:10,000, and a cutoff of 1:1000 will be used for our subsequent studies.

Early viral neutralization, with the ensuing prevention of the catastrophic immune response to viral damage, forms the basis for the infusion of high-titer CCP. Our expectation at protocol inception was to have access to patients early in the course of their disease. The reality, however, of conducting a clinical trial in the setting of an overwhelming influx of cases meant that most patients were not hospitalized until later in their course, during the inflammatory phase. We therefore conducted an analysis of the nonimmune patients, which included patients early in their course and patients unable to mount an immunity, such as immunocompromised patients. Understanding the kinetics of immune response to the virus is important and has been recently elegantly described. In a series of 23 patients with mild or severe disease (24), IgG Abs emerged at 10–15 days after onset of symptoms and were sustained for at least 6 weeks, and with a similar IgG response for both the mild and severe groups. Based on these kinetic descriptions, we can support that the presence of Abs on day 3 was from passive transfer and not time related. Interestingly, the same authors reported that most patients with severe disease still had viral shedding 30–40 days after onset of disease, bringing into question the neutralizing capability of those endogenous Abs (25). In our study, recipients demonstrated a high level of viral clearance at postinfusion days 10 and 30.

Track 3 represents a group of severely ill patients, either noninvasively or invasively mechanically ventilated, all with endogenous immune titers. Our management of patients with COVID-19 from mid-April 2020 through mid-June reserved invasive ventilation almost exclusively for patients failing noninvasive positive pressure ventilation measures. The clinical course and outcomes of critically ill patients with SARS-CoV-2 pneumonia have been previously reported (26, 27). In a series of 52 patients similar to our track 3 patients, receiving either invasive or noninvasive mechanical ventilation, 32 patients (61.5%) had died by day 28, and of the remaining 20 patients, only 8 patients (15.4%) were discharged (26). In our current study, track 3 patients had a day-30 discharge alive rate of 46.7% and a viral clearance of 86.7% at day 10 after treatment. This may support the position that passive transfer of antiviral titers may be of benefit even in patients with immunity.

The focus of most antiviral therapy has been early in the course of the disease. The track 2 day-30 discharge alive rate for patients was 88.9%, even though 22% of patients were immunocompromised either from cancer or transplantation, 100% had pneumonia, and 89% required oxygen supplementation. A recent randomized study evaluated the effect of CCP on the time to symptom improvement in severe COVID-19 disease (13). Patients were excluded if they had high titers of spike protein RBD–specific IgG Abs (≥1:640), leaving a similar patient population to our nonimmune or minimally immune patients (≤1:100–500). The median volume infused was 200 mL compared with 400–500 mL in our study. In this randomized study, the day-28 mortality was 15.7% for patients in the plasma group, with a discharge rate of 51%. Details of the plasma content or immunity transfer were not provided. However, there was a statistically significant increase (*P* < 0.001 at 72 hours) in the rate of viral negativity by RT-PCR in the plasma group but no difference in the primary outcome of time to clinical improvement. Moreover, this study was unfortunately limited by the small sample size.

Our study was limited by the lack of randomization to a control group, and the access to patients early in the disease course, where antiviral interventions are presumed to be of greatest impact. Our study was also not powered or designed to evaluate the optimal donor antiviral titer level, or the optimal dose of IgM and IgG to be infused. We are conducting a randomized study of CCP in high-risk patients with early-onset disease with the aim of reducing hospitalizations.

In conclusion, we aimed to better understand the clinical and laboratory effects of high-titer CCP in hospitalized patients with severe COVID-19 pneumonia. We found that the infusion of CCP was safe, effectively transferred titers, led to a high incidence of viral clearance, and did not preclude the development of endogenous immunity. The low rate of intubation and the survival at day 30 are encouraging and warrant further evaluation within the context of a randomized study.

## Methods

### Study design.

We conducted a single-institution prospective phase IIa clinical trial. The study was performed at Hackensack University Medical Center. Patients were included if they were 18 years of age or older and were hospitalized for the management of symptoms associated with a documented infection with SARS-CoV-2. Patients were excluded for a history of severe transfusion reactions, infusion of immunoglobulins with 30 days, aspartate transaminase or alanine aminotransferase greater than 10 times the upper limit of normal, requirement for vasopressors, and dialysis. Patients requiring intermittent vasopressors for sedation management were treated. The patient referral process was done by requests to a central research team. Any treating clinician could refer their patients to a central COVID-19 research basket requesting participation in this and other studies. A research nurse would perform an initial screen, and patients who appeared eligible for this CCP study were then approached by the study’s research nurses for final confirmation of eligibility and consenting purposes.

Prospective plasma donors were included if they were 18–60 years of age, had a history of a positive nasopharyngeal swab for COVID-19 or a positive Ab test, were at least 14 days from resolution of symptoms, had 1 subsequent negative swab, were found to have high titers of neutralizing Abs against SARS-CoV-2 (>1:500), and met institutional and FDA regulations for donation of blood products.

### Procedures.

Volunteer donors were recruited through advertising in the local community. Individuals who agreed to participate and gave informed consent were evaluated at the John Theurer Cancer Center, where they underwent a physical examination, completed a donor health questionnaire, had a nasopharyngeal swab for SARS-CoV-2, and had blood drawn for complete blood count and chemistry, infectious disease markers, and HLA Abs for female donors. These donors were then collected either at our facility or referred to our affiliated blood center. Plasma collected on site was distributed fresh in 500 mL bags, and plasma collected through our affiliated blood center was frozen in 200 mL bags. Collection at either site was based solely on availability.

The presence of SARS-CoV-2 neutralizing Abs was evaluated using the previously described COVID-19 ELISA protocol with RBD as capture antigen, using Goat anti–Human IgG (H+L) Secondary Ab, HRP (Thermo Fisher Scientific, catalog 31410) (26). High-titer sera were evaluated for virus neutralization in a viral cytopathic assay performed with Vero E6 cells at 100 times the tissue culture infection dose value. The assay using SARS-CoV-2 in Vero E6 cells was established under biosafety level 3 containment to assess intracellular inhibitory potencies of small molecules. Final assay conditions were 30,000 Vero E6 cells per well and virus at a MOI of 0.01–0.05 in 200 μL. The plates were incubated for 48 or 72 hours at 37°C and 5% CO_2_. Viral ToxGlo Luminescent Cell Viability Kit (Promega) was used to provide a semiquantitative measure of virus-infected cell viability. We also assessed the levels of IgM using the RBD antigen as per the IgG ELISA, with Goat anti–Human IgM Secondary Ab, HRP (Thermo Fisher Scientific, catalog 31415). Donors with a neutralizing IgG spike RBD greater than 1:500 were selected for plasma donation, with a preference for titers 1:1000–10,000 and greater than 1:10,000. Donors underwent plasmapheresis using the Trima Accel system for either a planned fresh infusion of 500 mL or cryopreservation in aliquots of 200 mL.

Recipients were referred by the clinical teams through the institutional COVID-19 research request process and were treated if eligible. A single infusion of CCP was administered at a rate less than 250 mL per hour. Premedication with diphenhydramine 25 mg i.v. and hydrocortisone 100 mg i.v. with or without acetaminophen was given. The use of fresh versus frozen plasma was based solely on the availability of product at the time of request. Exploratory blood work, including serology for anti-SARS-CoV-2 titers, was performed immediately preinfusion and on days 3, 10, 30, and 60 after treatment. SARS-CoV-2 testing by RT-PCR from nasopharyngeal or endotracheal tube secretions was performed on day 10 and if positive again on day 30. A 10 mL sample of plasma was collected at the bedside from the donor plasma bag immediately preinfusion for analysis.

For comparison, we evaluated the outcomes of patients treated for COVID-19 within our hospital network system. Data were collected from the electronic health records of patients hospitalized. Patients in the database were selected if SARS-CoV-2 RT-PCR tests were positive. Data were manually abstracted by nurses and physicians from the John Theurer Cancer Center as part of an unrelated Cancer Center Outcomes Division COVID-19 project. We selected all patients from this database with characteristics closest to the patients in our 2 treatment cohorts.

### Statistics.

It is important to note that at the time of the study’s statistical design in March 2020, the availability of outcomes data was more limited. Our statistical plan for this study included only patients ascribed to tracks 2 and 3. The primary endpoint for patients in track 2 was to evaluate the efficacy of CCP in reducing the rate of intubation. The primary objective for patients in track 3 was to evaluate the efficacy of CCP in reducing the mortality rate at day 30. The safety of CCP was also a primary objective. Secondary objectives for both groups included duration of hospitalization, OS, rate of virologic clearance by nasopharyngeal swab RT-PCR at days 10 and 30, impact of donor neutralizing Ab titer levels on the primary objectives, and recipient anti–SARS-CoV-2 titer levels preinfusion and on days 3, 10, 30, and 60. Comparison with patients in the COVID-19 database was not planned at inception and was performed post hoc.

We used a multistage design based on the sequential conditional probability ratio test, which is more efficient than Simon’s 2-stage design and has the flexibility of unplanned analysis (28). The design for each track had a type I error rate of 0.1 with statistical power of at least 0.8. The statistical design was based on the following hypothesis: for track 2 the null hypothesis assumed an intubation rate of 30%, and the alternative hypothesis was an intubation rate of less than or equal to 15%. The first stage analysis was after 12 patients. If 6 or more of the first 12 patients required mechanical ventilation to the therapy, there was less than 0.059% chance that the mechanical ventilation rate would be less than 30% should the study continue to enroll all 36 patients. However, if 0 of the first 12 patients required mechanical ventilation, it was certain that the trial would meet its goal even if we enrolled all 36 patients. For track 3, the null hypothesis assumed a mortality rate of at least 49% with an alternative hypothesis of less than or equal to 25%. The first stage analysis was after 6 patients, if 5 or more of the first 6 patients died, there would be a less than 0.091% chance that the mortality rate would be less than 49% should the study continue to enroll all 19 patients. However, if 0 of the first 6 patients died, it was certain that the trial would meet its goal even if we enrolled all 19 patients. In accordance with this statistical plan, track 3 enrolled a total of 15 patients before closing. The decision to accept or reject the null hypothesis was made based on interim data analysis in a 3-stage process. Descriptive statistics were used to characterize the baseline profile of the subjects and exploratory outcomes. Frequency and percentages were used for categorical variables; mean (SD) and median (IQR) were used for the continuous variables. Confidence intervals for the intubation and mortality rates, and virologic clearance at day 10 and 30, were calculated using exact binomial. Kaplan-Meier method was used for OS. Log-rank statistics were used to compare the OS between product types, donor titers, and pretreatment immunity. Cox proportional hazards model was used to assess the effect of infused plasma neutralizing titers on OS. Univariate test was performed to explore associations between exploratory outcomes and interested groups. Fisher’s exact test was used for categorical variables, and 2-tailed *t* test or 1-way ANOVA, or its nonparametric version, for the continuous variables based on the normalized of the data. A *P* value of less than 0.05 was considered significant. Statistical analyses were performed using SAS (version 9.4) and RStudio (version 0.99.902).

### Study approval.

The study is registered with ClinicalTrials.gov NCT04343755, FDA IND. Approval was obtained April 4, 2020, from the IRB of the Hackensack University Medical Center. All participants or their legally authorized representative provided written informed consent.

## Author contributions

The trial design and implementation were done by MLD, AI, SG, SR, KC, EB, JZ, KB, AU, LL, AR, MK, MV, RF, HS, DS, MG, TF, AG, AP, NB, LL, SS, SK, and DSP. Care of the patients was done by BB, CC, RS, AAK, SS, SD, DA, RG, ET, KR, SS, AG, ST, AM, MB, and PK. The statistical design and analysis were done by XG and MT. The laboratory and basic science were done by SP, SF, RK, and DSP.

## Figures and Tables

**Table 1:**
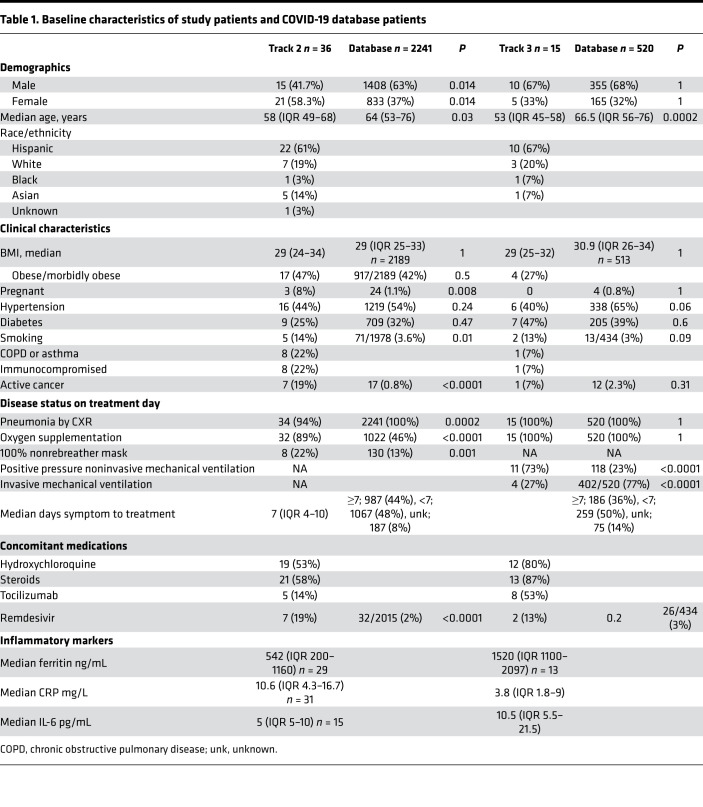
Baseline characteristics of study patients and COVID-19 database patients

**Table 2:**
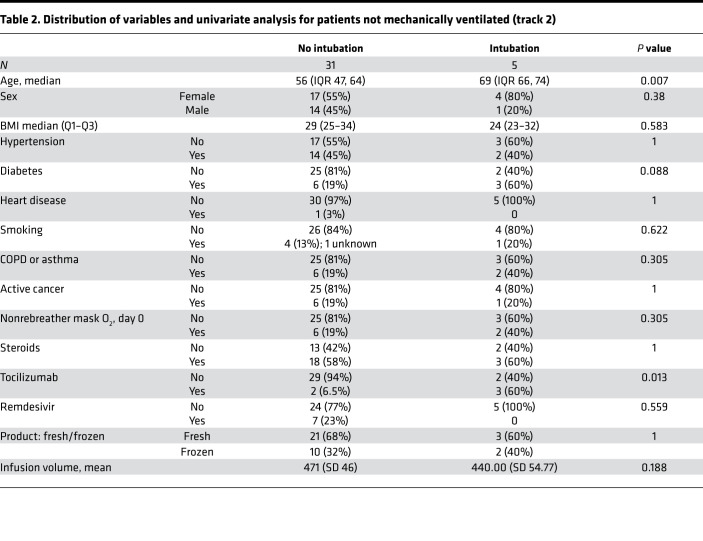
Distribution of variables and univariate analysis for patients not mechanically ventilated (track 2)

**Table 4:**
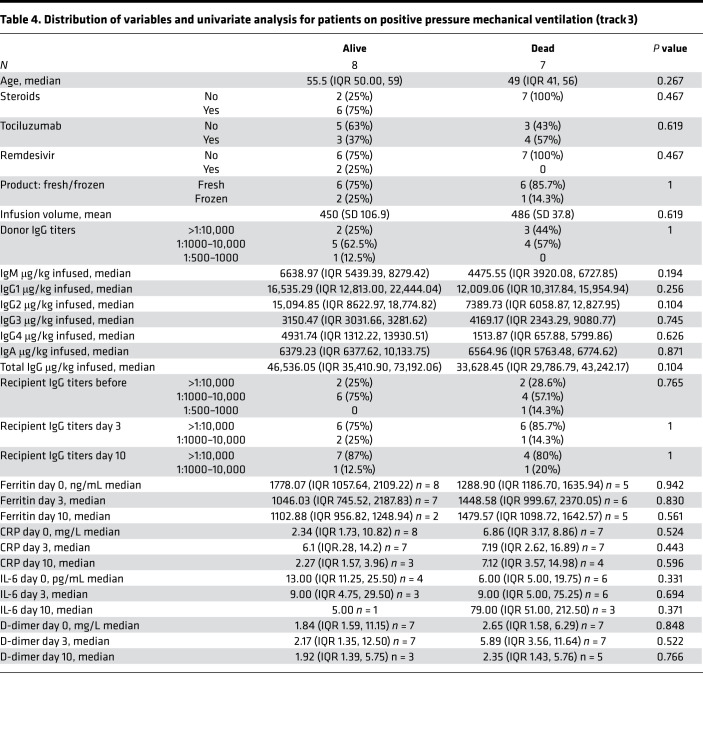
Distribution of variables and univariate analysis for patients on positive pressure mechanical ventilation (track 3)

**Table 5:**
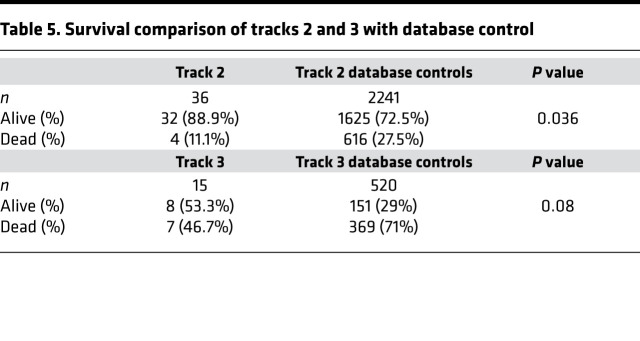
Survival comparison of tracks 2 and 3 with database control

**Table 3:**
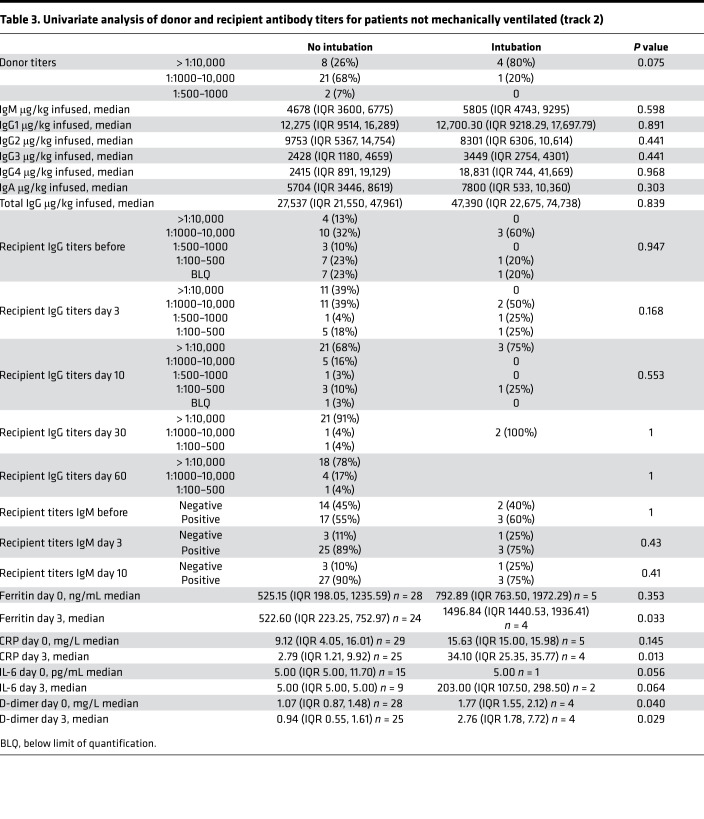
Univariate analysis of donor and recipient antibody titers for patients not mechanically ventilated (track 2)

## Version 1 02/11/2021

In-Press Preview

## Version 2 03/22/2021

Electronic publication
